# Trends, survival and regional control of sentinel lymph node biopsy versus axillary dissection in cN0 breast cancer: a multicenter cohort in China

**DOI:** 10.3389/fonc.2026.1809288

**Published:** 2026-06-24

**Authors:** Jiang Wu, Jiahui Xiang, Dongxu Ma, Heng Cao, Zizhao Guo, Lin Cong, Ruijie Zhou, Xinke Shi, Yuchen Liu, Yansong Huang, Jiaqi Liu, Xiang Wang

**Affiliations:** 1 National Cancer Center/National Clinical Research Center for Cancer/Cancer Hospital, Chinese Academy of Medical Sciences and Peking Union Medical College, Beijing, China; 2 Beijing Chaoyang Hospital Affiliated to Capital Medical University, Beijing, China

**Keywords:** axillary de-escalation, axillary lymph node dissection, clinically node-negative, pn1 disease, regional nodal recurrence, sentinel lymph node biopsy

## Abstract

**Background:**

Trials show that sentinel lymph node biopsy (SLNB) can replace completion axillary lymph node dissection (ALND) for selected cN0 patients without compromising survival but real-world evidence from non-Western settings especially on regional nodal control remains limited.

**Methods:**

We identified 53,758 female patients with cN0, pT1-3, pN0–1 breast cancer from the National Cancer Center Oncology Information Database (2013-2022). For pN1, survival analyses were restricted to 2017-2022. Propensity score matching and competing-risks models were employed to compare overall survival, breast cancer specific survival, and regional nodal recurrence (RNR) between SLNB and ALND.

**Results:**

SLNB use increased from 18.8% to 75.3% in pN0 and from 9.7% to 48.2% in pN1. In pN0, 5-year RNR was similar for SLNB versus ALND (1.06% vs 0.96%; adjusted SHR 1.03). In pN1, SLNB was associated with higher 5-year RNR after breast-conserving surgery (BCS) (4.99% vs 1.27%; adjusted SHR 4.44; P = 0.033), most pronounced for Ki-67 ≥30% (SHR 12.79; P = 0.012) and no special type histology (SHR 5.90; P = 0.017). In the mastectomy cohort, 5-year RNR did not differ between SLNB and ALND (3.01% vs 2.05%; P = 0.946; adjusted SHR 1.22), except among patients without endocrine therapy (SHR 6.28; P = 0.01).

**Conclusion:**

Axillary de-escalation has been widely adopted in China and appears oncologically safe for patients with pN0 disease and for those with pN1 disease undergoing mastectomy. In contrast, among patients with pN1 disease treated with breast-conserving surgery, SLNB was associated with a higher risk of regional nodal recurrence. When ALND is omitted, guideline-concordant adjuvant management may be important to maintain regional control.

## 1 Introduction

Breast cancer is one of the most commonly diagnosed cancers worldwide and remains a major threat to women’s health (1). Over the past three decades, surgery for early breast cancer has been progressively de-escalated. The introduction of sentinel lymph node biopsy (SLNB) in the 1990s reduced the extent of axillary surgery, shifting from routine axillary lymph node dissection (ALND) for clinically node-negative (cN0) patients to a more selective approach (2). With similar cancer control and lower complication rates (e.g., lymphedema and sensory loss), SLNB is the standard staging procedure for cT1-2N0 tumors (3). The 2025 update from the American Society of Clinical Oncology (ASCO) states that clinicians may offer SLNB for cT3–4 disease with a clinically negative axilla (low-certainty recommendation) (4), and the National Comprehensive Cancer Network (NCCN) lists SLNB as an axillary staging option in cT3-4N0 disease without an explicit contraindication (5). Additionally, landmark randomized trials have fundamentally redefined the standard of care for patients with limited nodal metastasis. The ACOSOG Z0011 trial demonstrated that ALND could be safely omitted without compromising survival in patients undergoing breast-conserving surgery (BCS) (6), while the AMAROS trial validated axillary radiotherapy as an equally effective but less morbid alternative to surgical clearance (7). Most recently, the SENOMAC trial extended these de-escalation principles, supporting the omission of ALND in patients with T1–3 tumors and one to two macrometastatic sentinel nodes, regardless of whether they undergo breast conservation or mastectomy (8).

Despite pivotal trials, evidence gaps remain. Although studies such as the Milan trial (9), ALMANAC (10), NSABP-B32 (11) and ACOSOG Z0010 (12) established the safety of SLNB, they used selective eligibility criteria and modest sample sizes in tightly controlled settings, limiting generalizability to routine care. Consequently, the implementation and performance of SLNB in routine real-world practice remain poorly described. Real-world practice often departs from trial protocols, and both over- and undertreatment occur, influenced by cost, access, and local capacity. Population registries suggest that SLNB performs similarly to—or better than—ALND in pathologic node-negative T1–2 disease (13, 14), while robust data in T3 settings are sparse, leaving the net benefit in this broader group unresolved (15). An N1 classification based on a positive sentinel node (N1[sn]) may not capture the true nodal burden once potential metastases in non-sentinel nodes are considered. Therefore, the prognostic difference between N1(sn) and N1 determined after axillary dissection in real-world care has direct implications for treatment selection (16, 17).

To clarify how axillary management is applied in routine care, we conducted a nationwide, multicenter hospital-based retrospective cohort study using the NCCOID. The primary objective of our investigation was two-fold: (1) to quantify national trends in axillary de-escalation; (2) long-term regional node control and survival associated with SLNB versus ALND in cN0, pN1 breast cancer in the contemporary era. We further sought to identify independent prognostic factors and characterize high-risk subgroups to inform future individualized treatment strategies.

## 2 Materials and methods

### 2.1 Database

The NCCOID is a longitudinal, electronic medical record-based oncology information system covering 1,422 monitoring hospitals across all 31 provinces in mainland China, encompassing more than 10 million patients from 2013 to the present. It is the largest cancer database in China, comprising 25 interlinked tables organized into 19 domains and spanning 1,509 variables. The NCCOID integrates multidimensional data, including demographics, inpatient and outpatient records, diagnostic tests, medications, treatments, and follow-up information. The NCCOID is linked to the national cause-of-death surveillance system maintained by the Chinese Center for Disease Control and Prevention, allowing identification of cause-specific mortality. The database is securely maintained in compliance with privacy regulations and has received approval from the Human Genetic Resources Administration of China. To protect patient privacy, all identifiable personal information, such as names and ID numbers, has been removed to ensure confidentiality in line with ethical and legal standards (18). This study was approved by the Independent Ethics Committee of the National Cancer Center/Cancer Hospital (approval number: 23/0263765). All procedures were performed in accordance with the Declaration of Helsinki and the International Council for Harmonisation Good Clinical Practice guidelines.

### 2.2 Study population

We identified women with primary breast cancer (ICD-10 C50) in the NCCOID from 2013-2022, excluding non-primary breast cancers (e.g., ICD-10 C79.806). ICD-10 codes were cross-checked against diagnostic text across multiple structured forms to improve diagnostic accuracy. Eligibility criteria were age 18–85 years; cN0 disease; accurate records of axillary surgery (SLNB or ALND); and pathologic stage T1-T3, N0-N1, M0. We required at least 12 months of potential follow-up from the date of definitive surgery, unless death occurred earlier. To reduce bias from treatment selection and differences in nodal assessment, cohort entry was defined by preoperative clinical staging rather than pathologic staging. The geographic distribution of the enrolled participants across China was shown in **Supplementary Figure 1**. In China, axillary surgery and adjuvant therapy are guided mainly by three national guidelines: the National Health Commission Breast Cancer Diagnosis and Treatment Guidelines, the China Anti-Cancer Association Breast Cancer Committee (CACA-CBCS) Guidelines, and the Chinese Society of Clinical Oncology (CSCO) Guidelines. In routine practice, cN0 status is determined by physical examination and axillary ultrasound before axillary surgery. SLNB was defined as removal of one or more sentinel nodes, the first nodes receiving lymphatic drainage from the primary tumor, whereas ALND involved level I–II dissection, typically with pathologic evaluation of 10 or more nodes. Patients were categorized into pN0 and pN1 cohorts according to the registry-based pathologic nodal classification in the NCCOID, which follows the American Joint committee on Cancer (AJCC) staging system (19). In this database, isolated tumor cells were classified as pN0, whereas pN1mi was classified as pN1. Within each cohort, patients were grouped by axillary procedure (SLNB or ALND). Patients who had SLNB followed by completion ALND during the same definitive surgical episode were classified in the ALND group, whereas the SLNB group included patients without a recorded completion ALND.

**Figure 1:**
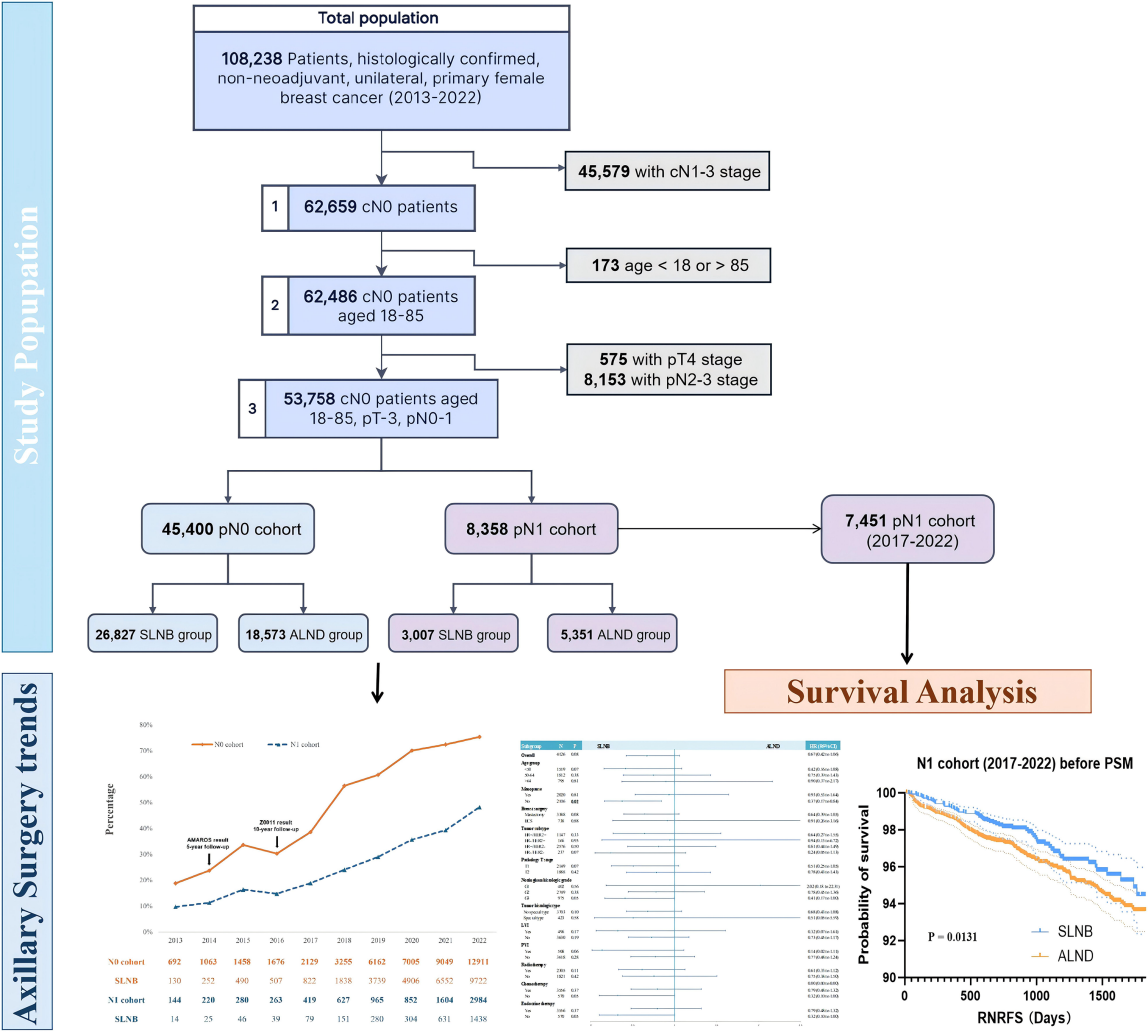
Flowchart of study population selection and cohort stratification. The study analyzed patients from the National Cancer Center Oncology Information Database (NCCOID) between 2013 and 2022, which were stratified into pN0 and pN1 cohorts. Survival analysis for the pN1 cohort was restricted to patients diagnosed between 2017 and 2022.

A cohort of 108,238 female patients with histologically confirmed, non-neoadjuvant, unilateral, primary breast cancer diagnosed between 2013 and 2022 was identified. We excluded 45,579 patients with cN1–3 stage and 173 patients aged <18 or >85 years. Additionally, 575 patients with pT4 stage and 8,153 patients with pN2–3 status were removed from the study. Ultimately, 53,758 cN0 patients (aged 18-85, pT1-3, pN0-1) remained for the further analysis. Notably, given the pivotal influence of the ACOSOG Z0011 trial on axillary management strategies for pN1 patients—particularly following the publication of its long-term follow-up results in 2016—we restricted the survival analysis of the pN1 cohort to the period between 2017 and 2022 (**Figure 1**).

### 2.3 Key variables

We abstracted data on demographics (age at diagnosis, year of diagnosis, menopausal status); tumor characteristics [pathologic T stage, histologic type, Nottingham histologic grade, molecular subtype, Ki-67 index, lymphovascular invasion (LVI), perineural invasion (PNI)]; breast surgery type; systemic therapy (chemotherapy, radiotherapy, and endocrine therapy); regional nodal recurrence (RNR); cause-specific mortality; and survival. The year of diagnosis was categorized into three calendar periods, including 2013-2016, 2017-2019, and 2020-2022, prespecified to reflect contemporary shifts in axillary practice. Age at diagnosis was categorized as <40, 40-49, 50-64, 65-74, and ≥75 years. Radiotherapy, chemotherapy, and endocrine therapy were coded as yes/no adjuvant treatments recorded after definitive surgery and before documented recurrence; treatments initiated after recurrence were not counted as adjuvant exposure. Radiotherapy may include whole-breast irradiation, postmastectomy radiotherapy, and regional nodal irradiation, but details of the treated fields were not available in the registry. Additionally, molecular subtype was defined by immunohistochemistry as hormone receptor-positive/HER2-negative (HR+/HER2-), hormone receptor-positive/HER2-positive (HR+/HER2+), hormone receptor-negative/HER2-positive (HR-/HER2+), or hormone receptor-negative/HER2-negative (HR-/HER2-). Patients were stratified by Ki-67 using a prespecified cutoff of 30%, chosen as a pragmatic threshold for defining a clearly high-proliferative subgroup despite recognized variation in Ki-67 thresholds across guidelines and clinical settings (20, 21). Data on RNR were abstracted from pathology or cytology reports, and details of ascertainment and time-to-event definitions are provided in the Outcomes section.

### 2.4 Outcomes

The study cutoff was December 31, 2023; follow-up was administratively censored on that date for all outcomes. Vital status and cause of death were obtained through linkage to the national cause-of-death surveillance system and categorized as alive, death from breast cancer, or death from other causes. The primary outcomes were overall survival (OS), breast cancer specific survival (BCSS), and regional nodal recurrence-free survival (RNRFS). OS was defined as the time from definitive surgery to death from any cause. BCSS was defined as the time from definitive surgery to death attributed to breast cancer, as recorded in the linked registry. RNR was defined as ipsilateral axillary or supraclavicular nodal recurrence confirmed by cytology or histology. Imaging-only suspicious nodal events without cytologic or histologic confirmation were not counted as RNR. Internal mammary nodal events were not included in the RNR endpoint. The event date for RNR was the date of the pathology or cytology report confirming recurrence. All time-to-event outcomes were measured from the date of definitive breast and/or axillary surgery. RNRFS was defined as the time from definitive surgery to the first RNR or death, whichever occurred first. Patients without an event were censored at the date of last follow-up.

### 2.5 Statistical analysis

Statistical analyses were conducted by a dedicated biostatistics team with expertise in oncology epidemiology and clinical data analysis (Beijing Yiyong Technology Co., Ltd.). Baseline characteristics were compared using t-tests for continuous variables and chi-squared tests (χ²) for categorical variables, as appropriate. Propensity score matching (PSM) was performed separately within the pN0 and pN1 cohorts using the MatchIt package. A 1:1 nearest-neighbor algorithm without replacement was applied on the logit of the propensity score. The propensity model included prespecified baseline clinicopathologic variables, including age, year of diagnosis, breast surgery type, tumor subtype, Ki-67 index, pathologic T stage, Nottingham histologic grade, histologic type, LVI, PNI, radiotherapy, chemotherapy, and endocrine therapy. Post-matching covariate balance was assessed using absolute standardized mean differences (SMDs), with values <0.10 considered indicative of adequate balance. Kaplan-Meier curves and log-rank tests were used to compare OS, BCSS, and RNRFS. Univariable and multivariable Cox proportional hazards models were used to estimate associations for OS, BCSS, and RNRFS, reported as hazard ratios (HRs) with 95% confidence intervals (CIs). The proportional hazards assumption was assessed using Schoenfeld residuals.

Competing-risks methods were applied to estimate the probability of RNR, treating death as a competing event. Cumulative incidence functions (CIFs) estimated 5-year RNR; differences were tested with Gray’s test. Fine-Gray subdistribution hazard models yielded subdistribution hazard ratios (SHRs) with 95% CIs, adjusted for prespecified covariates. Detailed radiotherapy fields were unavailable in this registry; thus, regional nodal irradiation (RNI) adequacy could not be directly adjusted. Subgroup analyses were exploratory and prespecified by key clinicopathological factors; results were interpreted cautiously. Analyses were conducted in R (version 4.3.3); two-sided α=0.05.

## 3 Results

### 3.1 Baseline characteristics of patients

Baseline characteristics of the 53,758 patients, stratified by axillary procedure, are summarized in **Table 1**. The cohort included 45,400 patients with pN0 and 8,358 with pN1. In pN0, 26,827 underwent SLNB and 18,573 underwent ALND; in pN1, 3,007 underwent SLNB and 5,351 underwent ALND. The baseline characteristics revealed statistically significant, yet generally modest, differences between the SLNB and ALND cohorts in both the pN0 and pN1 patient populations. In the pN0 group, patients managed with SLNB were, on average, slightly younger (53.8 vs 54.5 years; P<0.001), had marginally smaller tumors by both clinical and pathologic T stage (both P<0.001), and exhibited a minimal difference in Ki-67 index (25.34% vs 28.21%; P<0.001) compared to those who underwent ALND. Additionally, the SLNB procedure was more frequently utilized in recent years of diagnosis (P<0.001) and was strongly associated with BCS (26.7% vs 14.7%; P<0.001). Similar patterns were observed in the pN1 cohort: Patients in the SLNB group were also marginally younger (53.6 vs 54.5 years; P<0.001), more likely to undergo BCS (27.0% vs 10.8%; P<0.001), and presented with slightly smaller tumors (P<0.001) than those who received ALND.

**Table 1:** Baseline characteristic of enrolled patients before propensity score matching.

	pN0 Cohort			pN1 Cohort		
Characteristic	SLNB group(26827) no. (%)	ALND group(18573) no. (%)	P value	SLNB group(3007) no. (%)	ALND group(5351) no. (%)	P value
Year at diagnosis			<0.001			<0.001
2013-2016	1295 (4.8)	3594 (19.4)		124 (4.1)	783 (14.6)	
2017-2019	6070 (22.6)	5476 (29.5)		510 (17.0)	1501 (28.1)	
2020-2022	19462 (72.5)	9503 (51.2)		2373 (78.9)	3067 (57.3)	
Age						
Mean — yr	53.8 ± 11.3	54.5 ± 11.4	<0.001	53.6 ± 11.2	54.5 ± 11.3	<0.001
Median (range) — yr	53(19-85)	54(18-85)	<0.001	52(21-85)	54(21-85)	<0.001
Distribution — no. (%)			<0.001			0.001
<40 yr	2564 (9.6)	1610 (8.7)		297 (9.9)	447 (8.4)	
40–49 yr	7674 (28.6)	4964 (26.7)		867 (28.8)	1399 (26.1)	
50–64 yr	11376 (42.4)	8167 (44.0)		1294 (43.0)	2401 (44.9)	
65–74 yr	4173 (15.6)	2946 (15.9)		444 (14.8)	869 (16.2)	
≥75 yr	1040 (3.9)	886 (4.8)		105 (3.5)	235 (4.4)	
Menopause — no. (%)			<0.001			<0.001
Yes	13265 (49.4)	9899 (53.3)		1369 (45.5)	2782 (52.0)	
No	13562 (50.6)	8674 (46.7)		1638 (54.5)	2569 (48.0)	
Tumor Staging — no. (%)						
cT Stage			<0.001			<0.001
cT1	12675 (47.2)	7708 (41.5)		1191 (39.6)	1353 (25.3)	
cT2	12854 (47.9)	9400 (50.6)		1640 (54.5)	3429 (64.1)	
cT3	1298 (4.8)	1465 (7.9)		176 (5.9)	569 (10.6)	
pT Stage			<0.001			<0.001
pT1	17589 (65.6)	10984 (59.1)		1695 (56.4)	2436 (45.5)	
pT2	8758 (32.6)	7257 (39.1)		1258 (41.8)	2742 (51.2)	
pT3	480 (1.8)	332 (1.8)		54 (1.8)	173 (3.2)	
Breast surgery — no. (%)			<0.001			<0.001
BCS	7173 (26.7)	2727 (14.7)		812 (27.0)	579 (10.8)	
Mastectomy	19654 (73.3)	15846 (85.3)		2195 (73.0)	4772 (89.2)	
Tumor Histologic type — no. (%)			<0.001			<0.001
Invasive carcinoma, no special type	21559 (80.4)	16942 (91.2)		2466 (82.0)	4996 (93.4)	
Lobular carcinoma	1197 (4.5)	463 (2.5)		148 (4.9)	99 (1.9)	
mucinous carcinoma	1737 (6.5)	476 (2.6)		143 (4.8)	94 (1.8)	
medullary carcinoma	241 (0.9)	135 (0.7)		23 (0.8)	32 (0.6)	
metaplastic carcinoma	146 (0.5)	54 (0.3)		16 (0.5)	14 (0.3)	
Other	1947 (7.3)	503 (2.7)		211 (7.0)	116 (2.2)	
Nottingham histologic grade — no. (%)			<0.001			<0.001
Grade 1	2742 (10.2)	1397 (7.5)		289 (9.6)	439 (8.2)	
Grade 2	18341 (68.4)	12450 (67.0)		2053 (68.3)	3413 (63.8)	
Grade 3	5744 (21.4)	4726 (25.4)		665 (22.1)	1499 (28.0)	
Tumor subtype — no. (%)			0.019			<0.001
HR+/HER2+	6908 (25.8)	4630 (24.9)		741 (24.6)	1533 (28.6)	
HR+/HER2-	17200 (64.1)	12114 (65.2)		1998 (66.4)	3260 (60.9)	
HR-/HER2+	1036 (3.9)	643 (3.5)		111 (3.7)	206 (3.8)	
HR-/HER2-	1683 (6.3)	1186 (6.4)		157 (5.2)	352 (6.6)	
Ki-67 proliferation index						
Mean — %	25.34 ± 20.35	28.21 ± 21.21	<0.001	24.6 ± 19.2	30 ± 21.5	<0.001
Median (range) — %	20(0-99)	20(1-99)	<0.001	20(1-95)	25(1-99)	<0.001
Lymphovascular invasion — no. (%)			<0.001			<0.001
Yes	2067 (7.7)	2245 (12.1)		287 (9.5)	1115 (20.8)	
No	24760 (92.3)	16328 (87.9)		2720 (90.5)	4236 (79.2)	
Perineural invasion — no. (%)			<0.001			<0.001
Yes	2256 (8.4)	1895 (10.2)		359 (11.9)	831 (15.5)	
No	24571 (91.6)	16678 (89.8)		2648 (88.1)	4520 (84.5)	
Radiotherapy — no. (%)			<0.001			<0.001
Yes	8238 (30.7)	3837 (20.7)		1939 (64.5)	2115 (39.6)	
No	18595 (69.3)	14736 (79.3)		1068 (35.5)	3236 (60.4)	
Chemotherapy — no. (%)			<0.001			0.017
Yes	18224 (67.9)	13600 (73.2)		2363 (78.6)	4320 (80.8)	
No	8603 (32.1)	4973 (26.8)		644 (21.4)	1031 (19.2)	
Endocrine therapy — no. (%)			0.477			0.008
Yes	23100 (86.1)	16037 (86.3)		2634 (87.6)	4572 (85.5)	
No	3727 (13.9)	2536 (13.7)		373 (12.4)	779 (14.5)	

### 3.2 Trend towards de-escalation of axillary surgery

**Figure 2:**
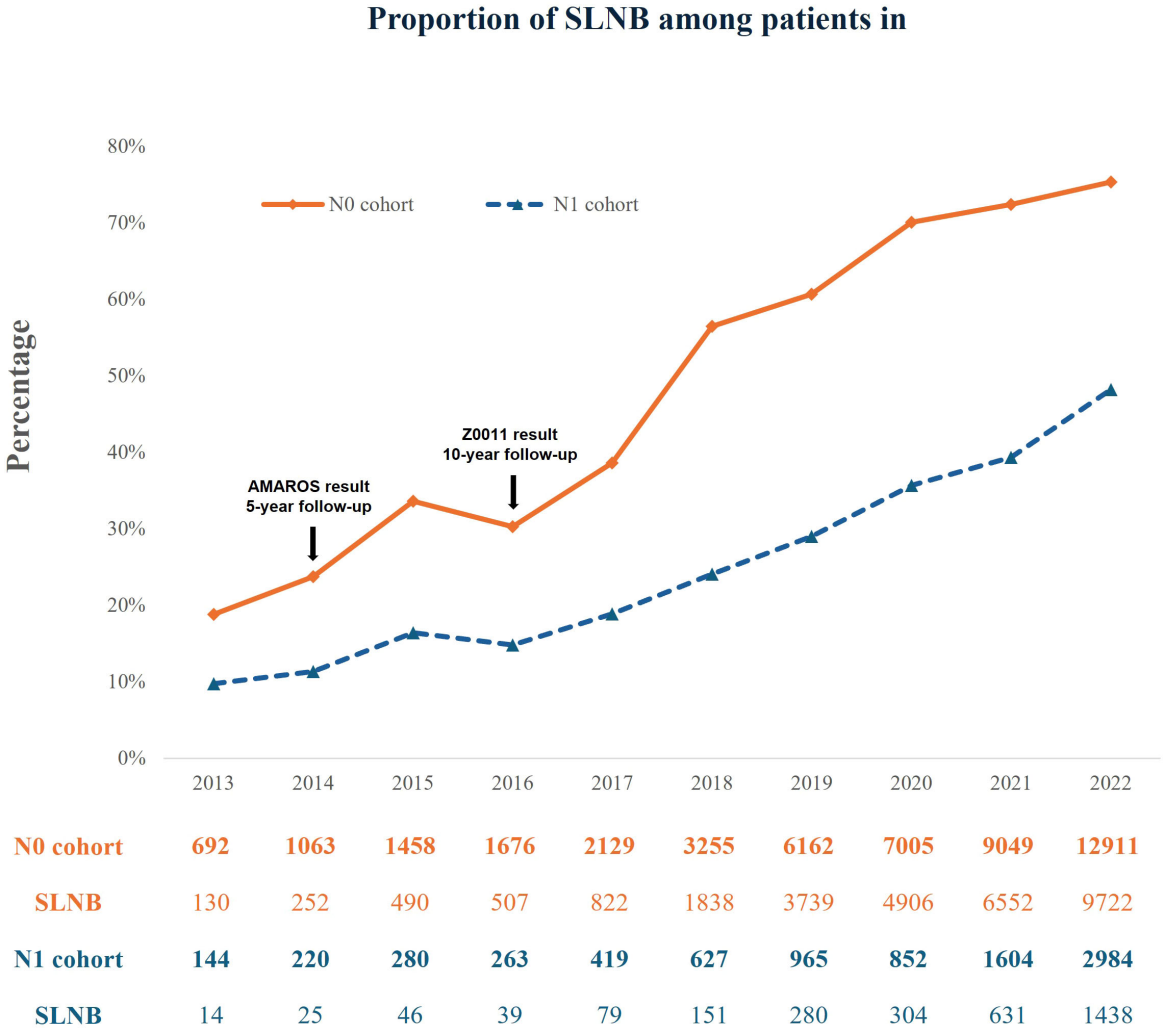
Temporal trends in the utilization of sentinel lymph node biopsy (SLNB) from 2013 to 2022. The graph illustrates the annual proportion of SLNB performed in the pN0 cohort (solid orange line) and the pN1 cohort (dashed blue line). Black arrows indicate the publication timing of pivotal trial follow-up results (AMAROS 5-year and ACOSOG Z0011 10-year) to contextualize shifts in clinical practice. The accompanying table lists the absolute numbers of patients and SLNB procedures for each year.

Between 2013 and 2022, there was a pronounced national trend toward the increased utilization of less extensive axillary surgery, in both the pN0 and pN1 cohorts (**Figure 2**). In the pN0 population, the proportion of patients undergoing SLNB demonstrated a substantial increase, rising from 18.8% to 75.3% over the decade. In the pN1 cohort, SLNB utilization also rose substantially, increasing from 9.7% to 48.2%.

### 3.3 Survival and multivariable Cox analysis

We restricted further survival analysis of the pN1 cohort to the period between 2017 and 2022. Kaplan-Meier curves compared 5-year OS, BCSS, and RNRFS between the SLNB and ALND groups. In **Supplementary Figure 2**, unadjusted 5-year OS, BCSS, and RNRFS were higher for SLNB than for ALND in both pN0 and pN1.

To address baseline imbalance, multivariable Cox proportional hazards models were fit. Variables associated with outcomes in univariable analyses entered the multivariable models (**Supplementary Figure 3**). In pN0, after adjustment, axillary procedure was not an independent predictor of OS, BCSS, or RNRFS. In pN1, SLNB was associated with more favorable OS [HR 0.64, 95% CI (0.43–0.95); P = 0.027] and BCSS [HR 0.45 )0.24–0.87); P = 0.020]. Associations with RNRFS were not statistically significant [HR 0.96 (0.73–1.27); P = 0.787].

### 3.4 Competing-risks model for mortality and recurrence

Because death is a significant event that may preclude the observation of RNR, we employed competing-risks methods to accurately estimate the association between the axillary procedure and RNR, treating death as a competing event. This analysis utilized CIFs with Gray’s test for unadjusted comparisons and Fine-Gray subdistribution hazard models to calculate the SHRs. Prespecified clinicopathologic covariates included axillary procedure, age, year of diagnosis, breast surgery type, tumor subtype, Ki-67 index, pathologic T stage, Nottingham histologic grade, histologic type, LVI, PNI, radiotherapy, chemotherapy, and endocrine therapy. Considering the established influence of both primary breast surgery and corresponding systemic treatment on RNR, we conducted subgroup analyses by stratifying the pN1 cohort into BCS and Mastectomy groups.

**Figure 3:**
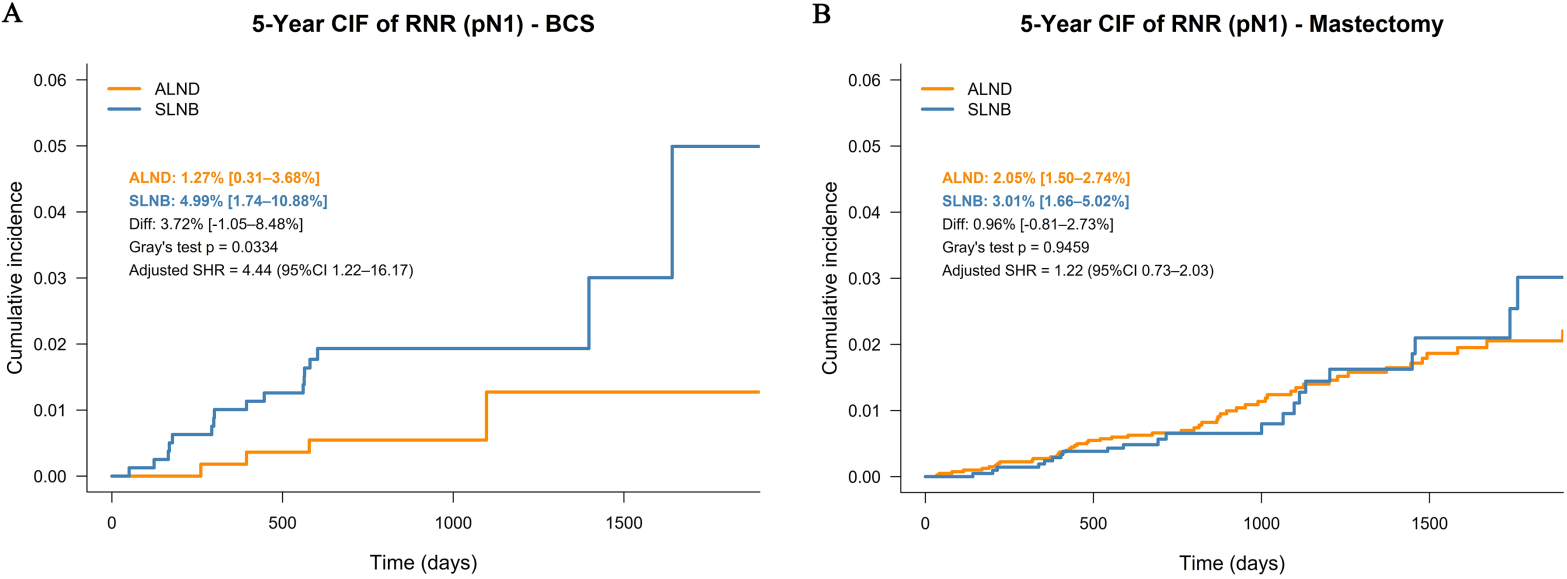
Cumulative incidence of regional nodal recurrence (RNR) in the pN1 cohort stratified by breast surgery type. The Competing-risks estimates of the 5-year cumulative incidence of RNR are shown for patients undergoing sentinel lymph node biopsy (SLNB, blue line) versus axillary lymph node dissection (ALND, orange line). The analysis is stratified into **(A)** the breast-conserving surgery (BCS) subgroup and **(B)** the mastectomy subgroup. Mortality was treated as a competing event. Insets summarize the 5-year cumulative incidence rates, absolute risk differences, P-values derived from Gray’s test, and multivariable-adjusted subdistribution hazard ratios (SHR) with 95% confidence intervals (CI).

Among patients who underwent BCS, 21 RNR events were observed. The 5-year cumulative incidence of RNR was significantly lower with ALND [1.27%; 95% CI, (0.31-3.68%)] compared to SLNB [4.99%; 95% CI, (1.74-10.88%)] (Gray’s test, P = 0.0334; **Figure 3A**). The multivariable Fine-Gray model confirmed this finding, showing that SLNB was associated with a significantly increased risk of RNR [adjusted SHR, 4.44; 95% CI, (1.22-16.17)]. By contrast, among patients who underwent mastectomy, 74 RNR events were observed, and the difference in RNR incidence between the two axillary procedures was negligible. The 5-year CIF of RNR was 2.05% [95% CI, (1.50-2.74%)] for ALND and 3.01% [95% CI, (1.66-5.02%)] for SLNB (Gray’s test, P = 0.9459; **Figure 3B**). Accordingly, SLNB was not associated with an increased risk of RNR in this subgroup [adjusted SHR, 1.22; 95% CI, (0.73-2.03)].

In pN0, 332 RNR events were observed. The 5-year cumulative incidence of RNR was 0.96% (95% CI 0.78-1.13) with ALND and 1.06% (95% CI 0.87-1.26) with SLNB (Gray’s test, P = 0.395; **Supplementary Figure 4**). The adjusted SHR from a multivariable Fine-Gray model was 1.03 (95% CI 0.80-1.32).

### 3.5 Patient matching and adjusted survival analyses

**Figure 4:**
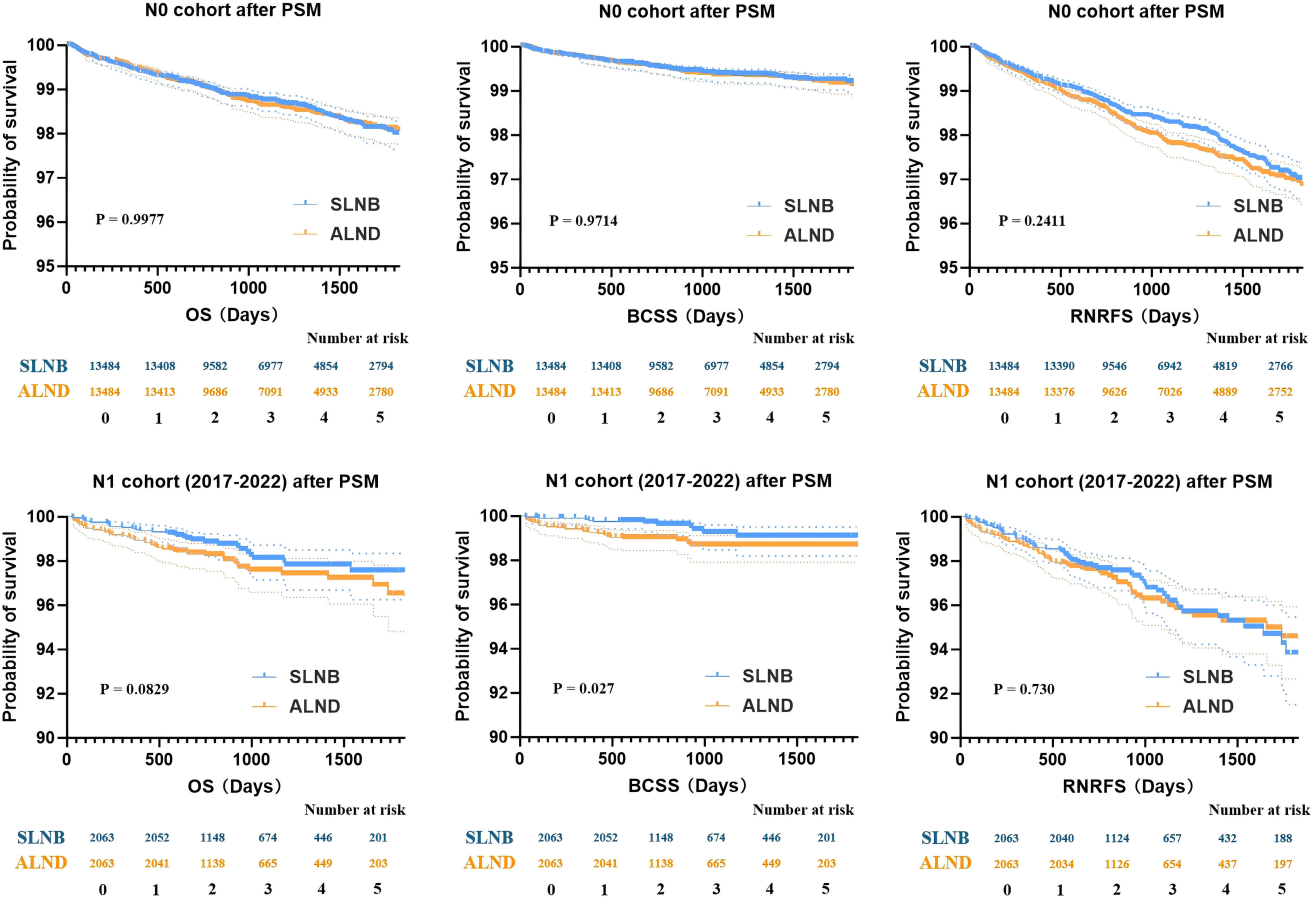
Kaplan-Meier survival outcomes after propensity score matching. The panels display overall survival (OS), breast cancer-specific survival (BCSS), and regional nodal recurrence-free survival (RNRFS) for the pN0 cohort (2013-2022, upper row) and the pN1 cohort (2017-2022, lower row). Solid lines compare sentinel lymph node biopsy (SLNB, blue) versus axillary lymph node dissection (ALND, orange), with dashed lines representing 95% confidence intervals. Log-rank P-values and numbers at risk are shown.

To reduce baseline confounding, we used PSM to generate matched cohorts of 26,968 pN0 and 4,126 pN1 patients (**Table 2**). Kaplan-Meier curves compared OS, BCSS, and RNRFS between SLNB and ALND (**Figure 4**). In pN1, SLNB was not associated with OS or RNRFS but was associated with better BCSS (P = 0.027). In pN0, after matching, SLNB and ALND showed no differences in OS, BCSS, or RNRFS.

**Table 2:** Baseline characteristic of enrolled patients after propensity score matching.

	pN0 Cohort (2013-2022)			pN1 Cohort (2017-2022)		
Characteristic	SLNB group (13484) no. (%)	ALND group (13484) no. (%)	P value	SLNB group (2063) no. (%)	ALND group (2063) no. (%)	P value
Year at diagnosis			0.013			0.650
2013-2016	1173 (8.7)	1073 (8.0)		NA	NA	
2017-2019	3807 (28.2)	3981 (29.5)		464 (22.5)	469 (22.7)	
2020-2022	8504 (63.1)	8430 (62.5)		1599 (77.5)	1594 (77.3)	
Age						
Mean — yr	54.51 ± 11.38	54.55 ± 11.51	0.791	53.95 ± 11.14	54.05 ± 11.39	0.787
Median (IQR) — yr	53 (47-63)	54 (46-63)	0.920	53 (46-62)	54 (46-63)	0.724
Distribution — no. (%)						
<40 yr	1148 (8.5)	1202 (8.9)	0.222	191 (9.3)	212 (10.3)	0.330
40–49 yr	3686 (27.3)	3593 (26.6)		577 (28.0)	539 (26.1)	
50–64 yr	5802 (43.0)	5863 (43.5)		915 (44.4)	897 (43.5)	
65–74 yr	2249 (16.7)	2179 (16.2)		300 (14.5)	336 (16.3)	
≥75 yr	599 (4.4)	647 (4.8)		80 (3.9)	79 (3.8)	
Menopause — no. (%)			0.558			
Yes	7180 (53.2)	7229 (53.6)		1002 (48.6)	1018 (49.3)	
No	6304 (46.8)	6255 (46.4)		1061 (51.4)	1045 (50.7)	
Tumor Staging — no. (%)						
cT Stage			0.335			<0.001
cT1	5889 (43.7)	5942 (44.1)		728 (35.3)	639 (31.0)	
cT2	6697 (49.7)	6597 (48.9)		1213 (58.8)	1220 (59.1)	
cT3	898 (6.7)	945 (7.0)		122 (5.9)	204 (9.9)	
pT Stage			0.646			0.854
pT1	8308 (61.6)	8351 (61.9)		1081 (52.4)	1068 (51.8)	
pT2	4956 (36.8)	4899 (36.3)		936 (45.4)	952 (46.1)	
pT3	220 (1.6)	234 (1.7)		46 (2.2)	43 (2.1)	
Breast surgery — no. (%) 0.087						
BCS	2600 (19.3)	2489 (18.5)		375 (18.2)	363 (17.6)	
Mastectomy	10884 (80.7)	10995 (81.5)		1688 (81.8)	1700 (82.4)	
Tumor Histologic type — no. (%)			0.784			0.931
Invasive carcinoma, no special type	12018 (89.1)	12093 (89.7)		1843 (89.3)	1860 (90.2)	
Lobular carcinoma	397 (2.9)	381 (2.8)		59 (2.9)	58 (2.8)	
mucinous carcinoma	87 (0.6)	82 (0.6)		62 (3.0)	57 (2.8)	
medullary carcinoma	51 (0.4)	44 (0.3)		13 (0.6)	13 (0.6)	
metaplastic carcinoma	450 (3.3)	426 (3.2)		9 (0.4)	6 (0.3)	
Other	481 (3.6)	458 (3.4)		77 (3.7)	69 (3.3)	
Nottingham histologic grade — no. (%)			0.604			0.990
Grade 1	1163 (8.6)	1126 (8.4)		200 (9.7)	202 (9.8)	
Grade 2	9081 (67.3)	9067 (67.2)		1374 (66.6)	1375 (66.7)	
Grade 3	3240 (24.0)	3291 (24.4)		489 (23.7)	486 (23.6)	
Tumor subtype — no. (%)			0.936			0.812
HR+/HER2+	3413 (25.3)	3405 (25.3)		561 (27.2)	586 (28.4)	
HR+/HER2-	8736 (64.8)	8715 (64.6)		1303 (63.2)	1273 (61.7)	
HR-/HER2+	497 (3.7)	501 (3.7)		82 (4.0)	84 (4.1)	
HR-/HER2-	838 (6.2)	863 (6.4)		117 (5.7)	120 (5.8)	
Ki-67 proliferation index						
Mean — %	27.11 ± 21.04	27.12 ± 20.76	0.970	26.36 ± 19.97	26.44 ± 20.24	0.900
Median (IQR) — %	20 (10-40)	20 (10-40)	0.446	20 (10-40)	20 (10-40)	0.933
Lymphovascular invasion — no. (%)			0.838			0.811
Yes	1336 (9.9)	1325 (9.8)		251 (12.2)	245 (11.9)	
No	12148 (90.1)	12159 (90.2)		1812 (87.8)	1818 (88.1)	
Perineural invasion — no. (%)			0.672			1.000
Yes	1215 (9.0)	1236 (9.2)		254 (12.3)	254 (12.3)	
No	12269 (91.0)	12248 (90.8)		1809 (87.7)	1809 (87.7)	
Radiotherapy — no. (%)			0.262			0.754
Yes	3311 (24.6)	3231 (24.0)		1158 (56.1)	1147 (55.6)	
No	10173 (75.4)	10253 (76.0)		905 (43.9)	916 (44.4)	
Chemotherapy — no. (%)			<0.001			0.620
Yes	9383 (69.6)	9754 (72.3)		1784 (86.5)	1772 (85.9)	
No	4101 (30.4)	3730 (27.7)		279 (13.5)	291 (14.1)	
Endocrine therapy — no. (%)			0.143			0.620
Yes	11659 (86.5)	11575 (85.8)		1784 (86.5)	1772 (85.9)	
No	1825 (13.5)	1909 (14.2)		279 (13.5)	291 (14.1)	

### 3.6 Subgroup analysis

As depicted in **Supplementary Figure 5**, regarding OS, SLNB was associated with better survival in the premenopausal and Nottingham grade G3 subgroups of pN1 cohort. For BCSS, trends favoring better survival with SLNB were observed in the premenopausal, mastectomy, Nottingham grade G2, no special type histology, LVI-negative, and receiving radiotherapy subgroups. Conversely, for RNRFS, estimates tended to favor ALND in the BCS subgroup.

**Figure 5:**
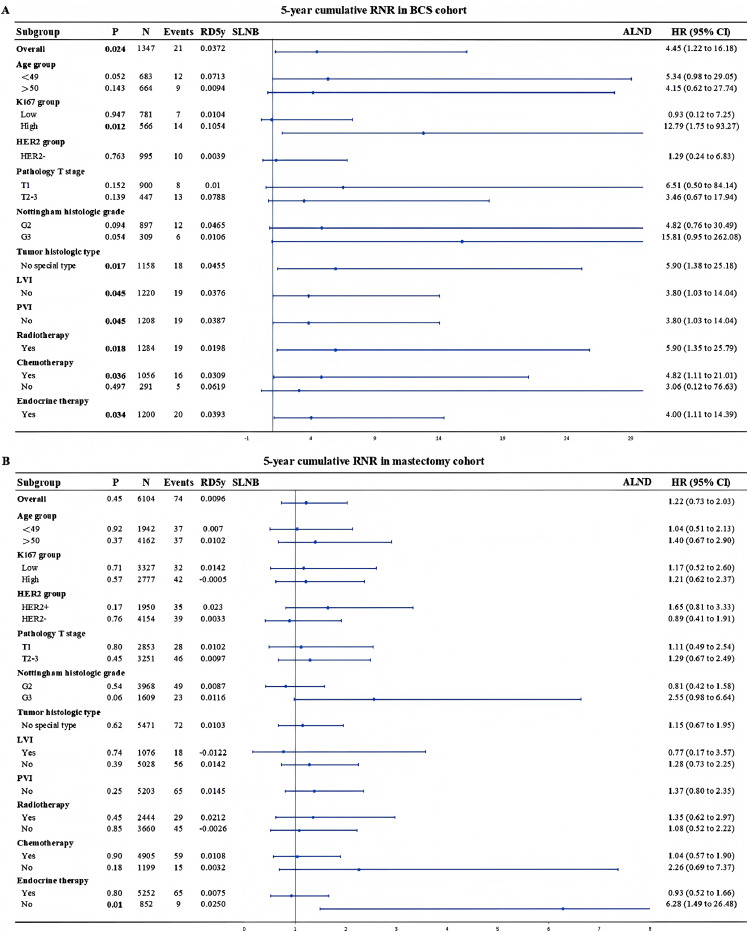
Subgroup analyses of regional nodal recurrence (RNR) in the pN1 cohort stratified by breast surgery type. Forest plots illustrate the hazard ratios (HR) for RNR comparing sentinel lymph node biopsy (SLNB) versus axillary lymph node dissection (ALND) among patients undergoing **(A) (B)** mastectomy. The analysis is stratified by key clinicopathological characteristics. Point estimates represent the HRs, with error bars indicating 95% confidence intervals (CI). The adjacent columns detail the subgroup-specific P-values, total patient numbers (N), event counts, 5-year risk differences (RD5y), and numerical HRs (95% CI).

Prespecified subgroup analyses were conducted in the pN1 cohort to evaluate effect modification by breast surgery type (**Figure 5**). The absolute 5-year difference in cumulative RNR (Δ5y RNR), defined as the 5-year cumulative incidence of RNR in the SLNB group minus that in the ALND group, is presented together with the SHR to describe both absolute and relative differences between groups. In the BCS cohort, the overall Δ5y RNR was 3.72%. SLNB was associated with a higher risk of 5-year RNR compared with ALND in patients with high Ki-67 (≥30%) (Δ5y 10.54%; SHR 12.79, 95% CI 1.75-93.27; P = 0.012) and in those with no special type histology (Δ5y 4.55%; SHR 5.90, 95% CI 1.38-25.18; P = 0.017). In patients who did not receive chemotherapy, the absolute 5-year difference in cumulative RNR was 6.19%; however, the adjusted association did not reach statistical significance. In the mastectomy cohort, the overall Δ5y RNR was 0.96%. The only subgroup showing a significantly higher risk with SLNB was patients who did not receive postoperative endocrine therapy (Δ5y 2.50%; SHR 6.28, 95% CI 1.49-26.48; P = 0.01), whereas the remaining subgroups showed no statistically significant differences.

## 4 Discussion

This nationwide multicenter study provides a comprehensive evaluation of trial-based axillary de-escalation strategies within a large, non-Western healthcare system. Our results confirm a paradigm shift toward axillary preservation in China over the past decade, demonstrating that this practice does not compromise overall survival in the broad population. Crucially, our mastectomy-dominant cohort bridges a significant knowledge gap between controlled RCTs and routine clinical practice (6, 7, 11, 22). While trials have established the safety of SLNB in selected populations, our real-world data expose a critical implementation challenge: in the absence of strict protocol adherence, simple surgical subtraction—omitting ALND without ensuring compensatory multimodal therapy—is associated with an increased risk of regional recurrence in patients undergoing BCS.

Although our temporal analysis confirms a clear trend toward axillary de-escalation, with ALND rates in pN0 patients declining to 24.7% by 2022, this persistence of dissection highlights a divergence from international consensus (23, 24). We interpret this residual reliance on ALND not as a statistical artifact, but as a genuine implementation gap driven by systemic barriers. Primarily, the logistical infrastructure required for standard-of-care SLNB, specifically the availability of radioisotopes and intraoperative frozen section pathology, remains unevenly distributed across regional centers (25). Secondarily, clinical translation is impeded by practice inertia, where historical quality metrics equating extensive dissection with superior surgical rigor continue to influence decision-making.

While neoadjuvant therapy is now standard for clinically node-positive disease, upfront surgery remains the predominant approach for most patients presenting with cN0 status in China and worldwide. For this prevalent cohort, optimizing the extent of axillary intervention, specifically the strategic interplay between surgical clearance and RNI, remains a pivotal area of debate. This is particularly critical for patients identified with sentinel lymph node metastasis, where defining the appropriate boundary of de-escalation is essential for balancing oncologic control with morbidity. Overall, our survival outcomes align with those reported in landmark trials and previous observational studies (13, 16, 26). The observed BCSS benefit among pN1 patients undergoing SLNB warrants cautious interpretation due to confounding by indication. Clinicians preferentially select SLNB for patients with favorable prognostic features, including younger age, smaller tumor burden, and luminal subtypes, while reserving ALND for high-risk presentations. Consequently, residual selection bias driven by unmeasured biological variables likely persists even after PSM.

Crucially, within the pN1 cohort we observed a clear divergence by breast surgery type: among patients undergoing breast-conserving surgery, SLNB was associated with a higher risk of regional nodal recurrence. This observation differs from the non-inferiority reported in ACOSOG Z0011. One possible explanation relates to differences in radiotherapy delivery rather than surgical technique alone. In ACOSOG Z0011, standard whole-breast irradiation using high tangential fields may have provided incidental coverage of axillary levels I and II, potentially contributing to regional control (6, 27). In contemporary real-world practice, however, more conformal treatment approaches may reduce this incidental axillary dose. As a result, variation in adjuvant treatment delivery after omission of ALND could partly influence regional control. In our study, the increased risk of RNR associated with SLNB was also observed among patients receiving adjuvant systemic therapy, suggesting that systemic therapy alone may not fully compensate for residual regional risk in some patients. This interpretation is broadly consistent with the rationale underlying trials such as AMAROS and SENOMAC (7, 8, 28), in which de-escalation of axillary surgery was evaluated in the context of protocol-directed adjuvant treatment. At the same time, differences across trials in endpoint definitions and event rates may make small but clinically relevant differences in nodal control more difficult to detect (29, 30). Because radiotherapy was recorded only as a binary variable in our registry, we were unable to directly assess the contribution of specific treatment fields, including regional nodal irradiation, to the observed differences in recurrence risk. Therefore, any interpretation regarding the role of RNI should be considered cautious and hypothesis-generating rather than confirmatory.

In contrast to the findings in the breast-conserving setting, in the mastectomy cohort SLNB was not associated with an increased risk of regional nodal recurrence compared with ALND. This finding is broadly consistent with the contemporary de-escalation paradigm and may help extend the evidence base beyond the population included in ACOSOG Z0011 (6), while also being in line with the recent SENOMAC trial (8). One possible explanation for the difference between the mastectomy and breast-conserving surgery cohorts is variation in adjuvant treatment patterns in routine practice. In some mastectomy-treated patients with positive sentinel nodes in whom ALND is omitted, postoperative radiotherapy may include regional nodal coverage, with or without chest wall irradiation (31, 32), which could partly compensate for the omission of surgical axillary clearance. However, because detailed radiotherapy field information was unavailable in our registry, this interpretation should be considered cautious and hypothesis-generating rather than confirmatory.

Our study has several limitations. First, radiotherapy was recorded only as a binary variable, and the registry did not provide detailed information on treatment fields. Therefore, we could not distinguish whole-breast irradiation, chest wall irradiation, and regional nodal irradiation, nor could we directly evaluate the association between specific radiotherapy fields and regional nodal recurrence. Second, the registry-based pN1 category could not be further separated into pN1mi and macrometastatic pN1 disease. Because these entities may differ clinically and biologically, this limitation may have introduced heterogeneity into the pN1 cohort. Third, the number of examined lymph nodes and positive lymph nodes by axillary procedure group were not reliably available in the registry, which limited a more granular comparison of nodal burden between the SLNB and ALND groups. Additionally, in the mastectomy cohort, non-receipt of endocrine therapy was identified as a predictor of higher recurrence risk; however, this subgroup likely included both patients with HR- tumors and HR+ patients who did not undergo treatment. Because of the limited sample size and small number of recurrence events after stratification, we could not reliably disentangle these possibilities.

## 5 Conclusions

In conclusion, this nationwide study documents the widespread adoption of axillary de-escalation in China over the past decade. Our findings support the oncologic safety of SLNB for patients with pN0 disease and for pN1 patients undergoing mastectomy. However, the higher risk of RNR observed among pN1 patients treated with BCS was mainly driven by the subgroups with Ki-67 ≥30% and no special type histology, and an increased RNR risk was also observed among mastectomy-treated pN1 patients who did not receive postoperative endocrine therapy. Given the observational nature of this study and the limited granularity of treatment data, these subgroup findings should be interpreted cautiously. Overall, our results suggest that when ALND is omitted, careful adherence to guideline-concordant adjuvant management may be important to maintain regional control.

## Data Availability

The original contributions presented in the study are included in the article/**Supplementary Material**. Further inquiries can be directed to the corresponding author.

## Supplementary material

Supplementary Figure S1 Geographic distribution of enrolled participants. The pie chart illustrates the proportional contribution of the study population from six major geographical regions across China within the National Cancer Center Oncology Information Database (NCCOID).

Supplementary Figure S2 Unadjusted Kaplan-Meier survival outcomes. The panels display overall survival (OS), breast cancer-specific survival (BCSS), and regional nodal recurrence-free survival (RNRFS) for the pN0 cohort (upper row) and pN1 cohort (2017–2022, lower row) prior to propensity score matching. Solid lines compare sentinel lymph node biopsy (SLNB, blue) versus axillary lymph node dissection (ALND, orange), with dashed lines representing 95% confidence intervals. Log-rank P-values and numbers at risk are shown.

Supplementary Figure S3 Multivariable Cox regression analysis of survival outcomes prior to propensity score matching. Forest plots illustrate adjusted hazard ratios (HR) for overall survival (OS, top row), breast cancer-specific survival (BCSS, middle row), and regional nodal recurrence-free survival (RNRFS, bottom row). The analysis is stratified by nodal status: the pN0 cohort (left column) and the pN1 cohort (2017–2022, right column). Error bars represent 95% confidence intervals (CI), with adjacent columns detailing patient counts, HRs, and P-values.

Supplementary Figure S4 Cumulative incidence of regional nodal recurrence (RNR) in the pN0 cohort. Competing-risks curves compare sentinel lymph node biopsy (SLNB, blue) versus axillary lymph node dissection (ALND, orange), treating death as a competing event. Insets display 5-year incidence rates, Gray’s test P-values, and multivariable-adjusted subdistribution hazard ratios (SHR).

Supplementary Figure S5 Subgroup analyses of survival outcomes in the matched pN1 cohort. Forest plots depict hazard ratios (HR) comparing sentinel lymph node biopsy (SLNB) versus axillary lymph node dissection (ALND) for **(A)** overall survival (OS), **(B)** breast cancer-specific survival (BCSS), and **(C)** regional nodal recurrence-free survival (RNRFS).

## Glossary

SLNB: Sentinel lymph node biopsy
ALND: axillary lymph node dissection
cN0: clinically node-negative
ASCO: American Society of Clinical Oncology
NCCN: National Comprehensive Cancer Network
N1[sn]: a positive sentinel node
pNI: pathologically node-positive
CACA-CBS: China Anti-Cancer Association Breast Cancer Committee
CSCO: Chinese Society of Clinical Oncology
LVI: lymphovascular invasion
PNI: perineural invasion
RNR: regional nodal recurrence
HR+: hormone receptor-positive
HR-: hormone receptor-negative
HER2+: HER2-positive
HER2-: HER2-negative
OS: overall survival
BCSS: breast cancer specific survival
RNRFS: regional nodal recurrence-free survival
PSM: Propensity score matching
HRs: hazard ratios
Cis: confidence intervals
CIFs: Cumulative incidence functions
SHRs: subdistribution hazard ratios
RNI: regional nodal irradiation
BCS: breast-conserving surgery
RCTs: randomized controlled trials
Δ5y RNR: absolute 5-year difference in cumulative RNR
pN0: node-negative disease
pN1: one to three positive nodes
SLNB: sentinel lymph node biopsy
ALND: axillary lymph node dissection
yr: year
cT stage: clinical T stage
pT stage: pathologic T stage
BCS: breast-conserving surgery
HR+: hormonal receptor positive
HR-: hormonal receptor negative
HER2+: human epidermal growth factor receptor 2 positive
HER2-: human epidermal growth factor receptor 2 negative
