# Correction: Trends, survival and regional control of sentinel lymph node biopsy versus axillary dissection in cN0 breast cancer: a multicenter cohort in China

**DOI:** 10.3389/fonc.2026.1919792

**Published:** 2026-07-29

**Authors:** Jiang Wu, Jiahui Xiang, Dongxu Ma, Heng Cao, Zizhao Guo, Lin Cong, Ruijie Zhou, Xinke Shi, Yuchen Liu, Yansong Huang, Jiaqi Liu, Xiang Wang

**Affiliations:** 1 National Cancer Center/National Clinical Research Center for Cancer/Cancer Hospital, Chinese Academy of Medical Sciences and Peking Union Medical College, Beijing, China; 2 Beijing Chaoyang Hospital Affiliated to Capital Medical University, Beijing, China

**Keywords:** axillary de-escalation, axillary lymph node dissection, clinically node-negative, pn1 disease, regional nodal recurrence, sentinel lymph node biopsy

Author “Jiaqi Liu” was erroneously omitted as corresponding author. The correct corresponding authors are Jiaqi Liu (j.liu@cicams.ac.cn) and Xiang Wang (xiangw@vip.sina.com).

The original version of this article has been updated.

