# Supplementary figures and images for: Trends, survival and regional control of sentinel lymph node biopsy versus axillary dissection in cN0 breast cancer: a multicenter cohort in China
Source: Front Oncol. 2026 Jun 24;16:1809288. doi: 10.3389/fonc.2026.1809288 (PMC13341454; doi:10.3389/fonc.2026.1809288)

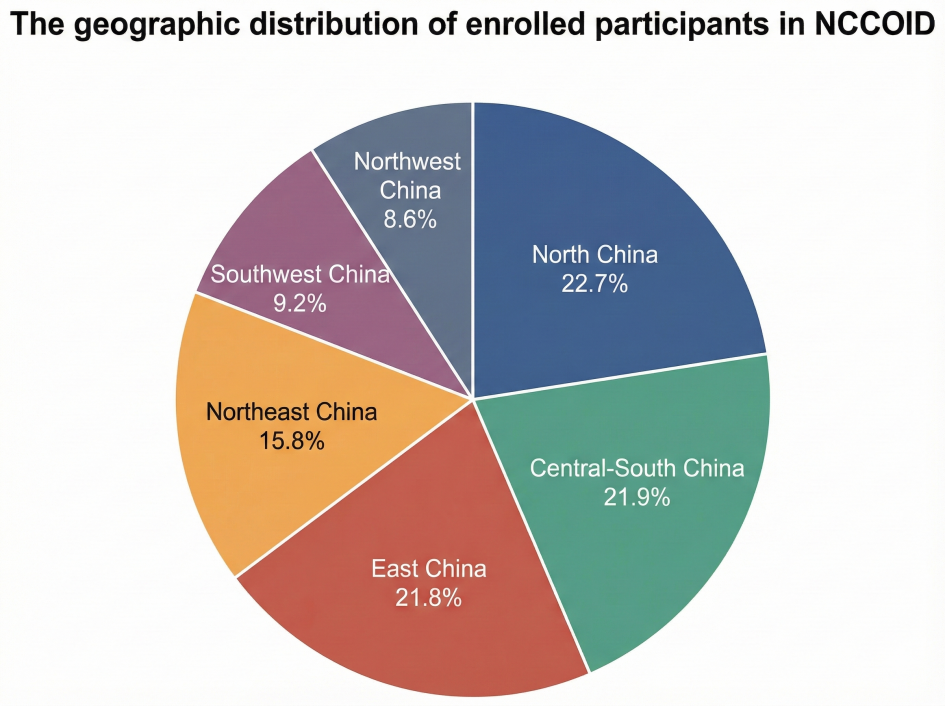

Supplement: Supplementary Figure S1 — Geographic distribution of enrolled participants. The pie chart illustrates the proportional contribution of the study population from six major geographical regions across China within the National Cancer Center Oncology Information Database (NCCOID). [file Image1.png]

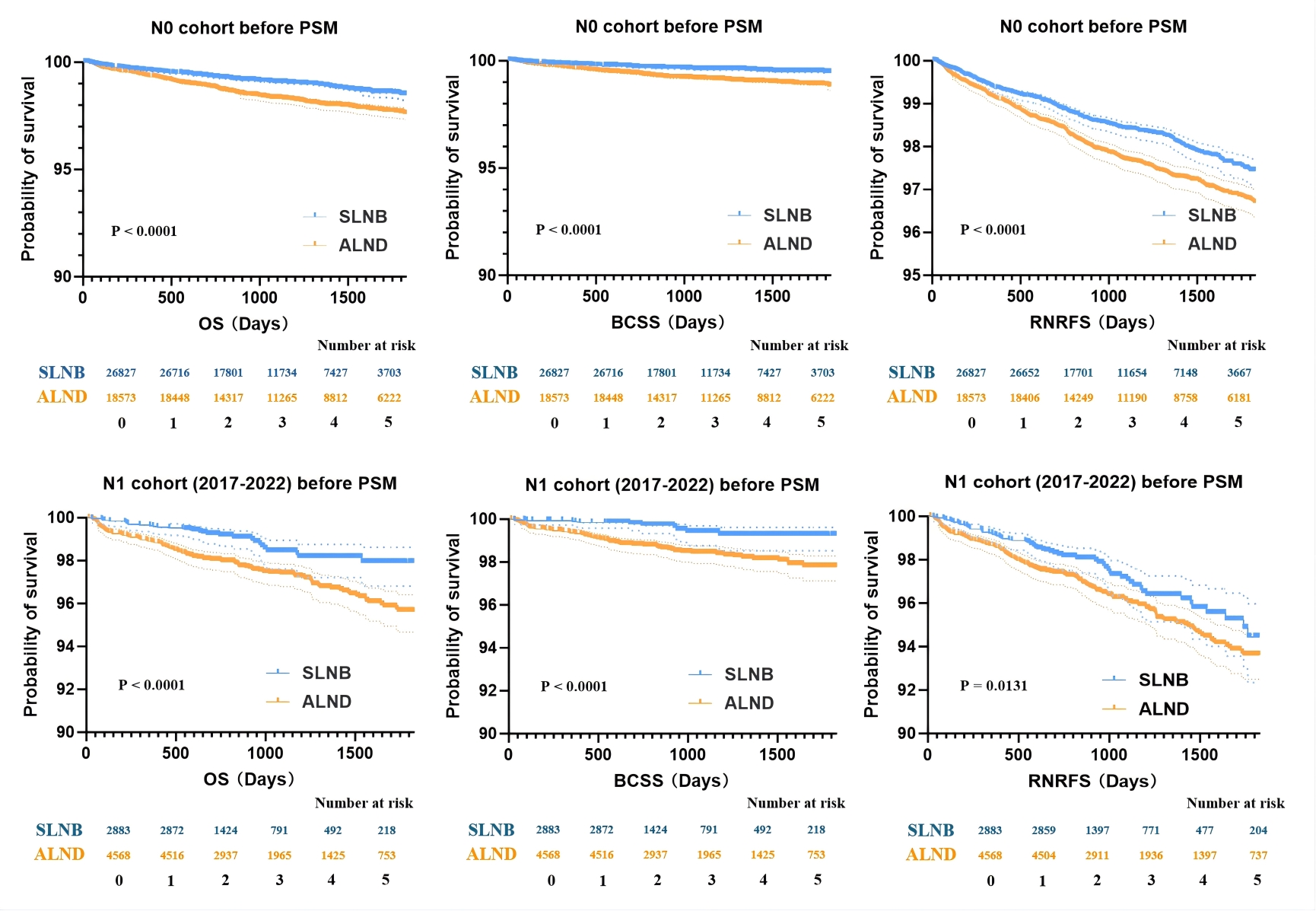

Supplement: Supplementary Figure S2 — Unadjusted Kaplan-Meier survival outcomes. The panels display overall survival (OS), breast cancer-specific survival (BCSS), and regional nodal recurrence-free survival (RNRFS) for the pN0 cohort (upper row) and pN1 cohort (2017–2022, lower row) prior to propensity score matching. Solid lines compare sentinel lymph node biopsy (SLNB, blue) versus axillary lymph node dissection (ALND, orange), with dashed lines representing 95% confidence intervals. Log-rank P-values and numbers at risk are shown. [file Image2.jpeg]

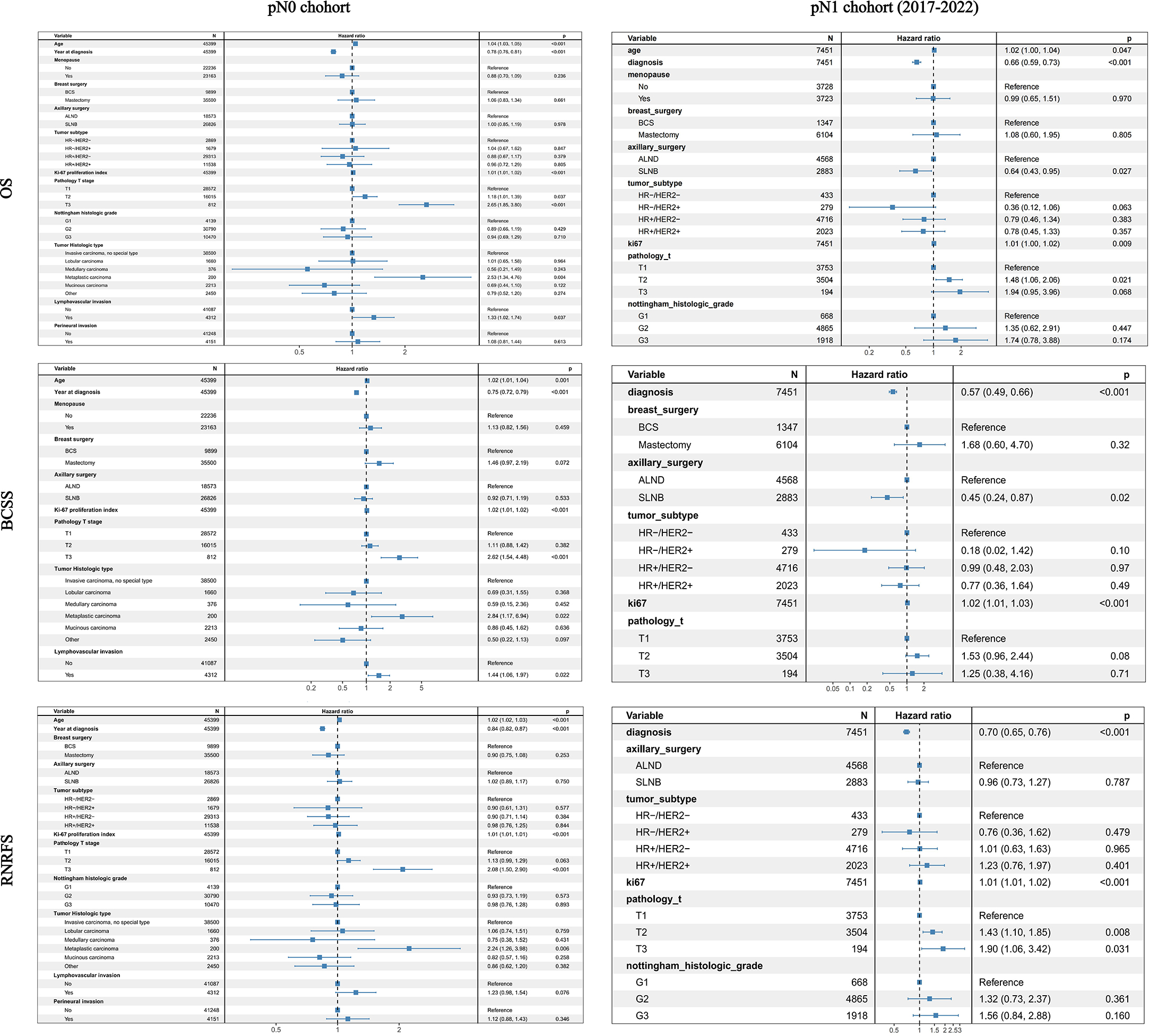

Supplement: Supplementary Figure S3 — Multivariable Cox regression analysis of survival outcomes prior to propensity score matching. Forest plots illustrate adjusted hazard ratios (HR) for overall survival (OS, top row), breast cancer-specific survival (BCSS, middle row), and regional nodal recurrence-free survival (RNRFS, bottom row). The analysis is stratified by nodal status: the pN0 cohort (left column) and the pN1 cohort (2017–2022, right column). Error bars represent 95% confidence intervals (CI), with adjacent columns detailing patient counts, HRs, and P-values. [file Image3.jpeg]

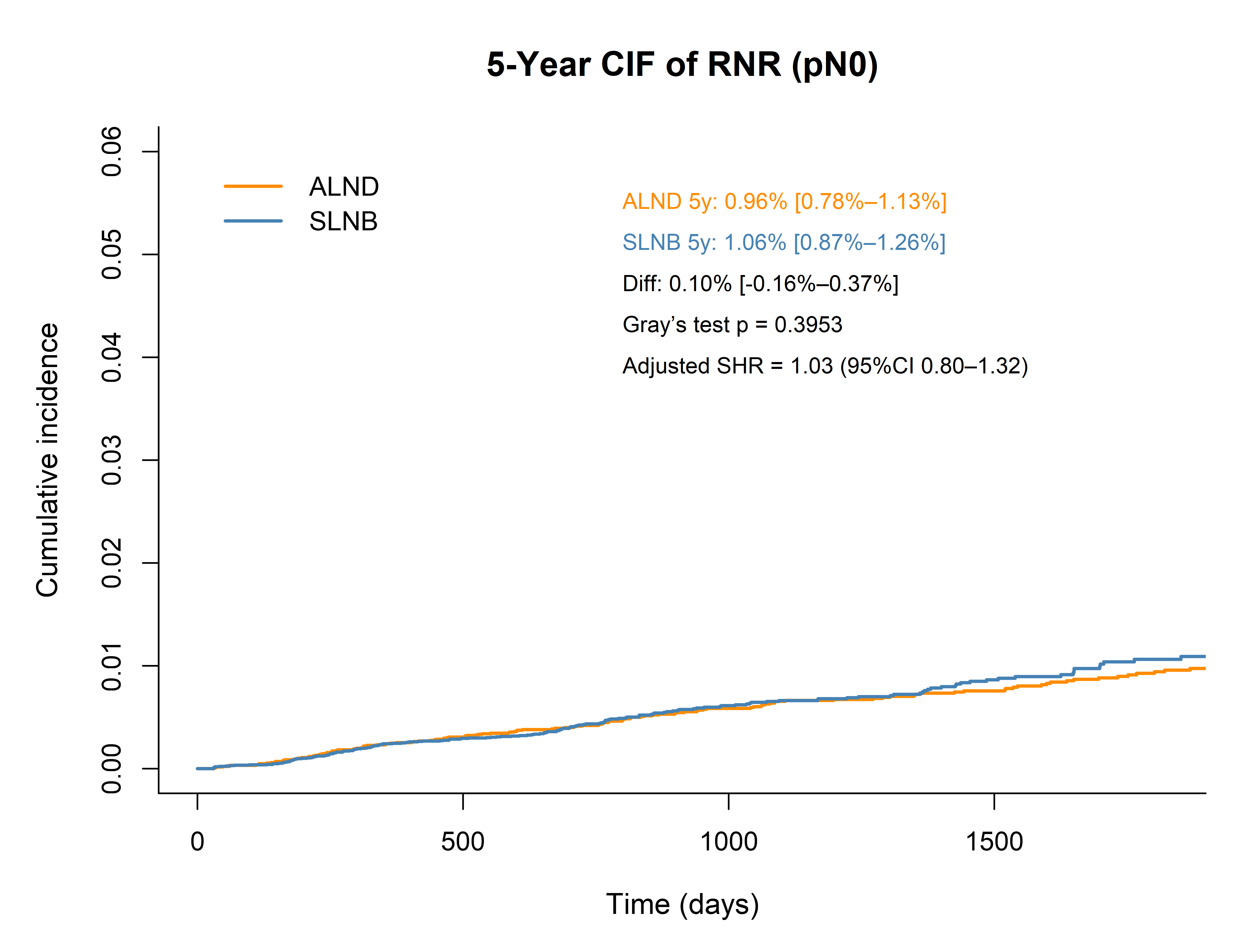

Supplement: Supplementary Figure S4 — Cumulative incidence of regional nodal recurrence (RNR) in the pN0 cohort. Competing-risks curves compare sentinel lymph node biopsy (SLNB, blue) versus axillary lymph node dissection (ALND, orange), treating death as a competing event. Insets display 5-year incidence rates, Gray’s test P-values, and multivariable-adjusted subdistribution hazard ratios (SHR). [file Image4.png]

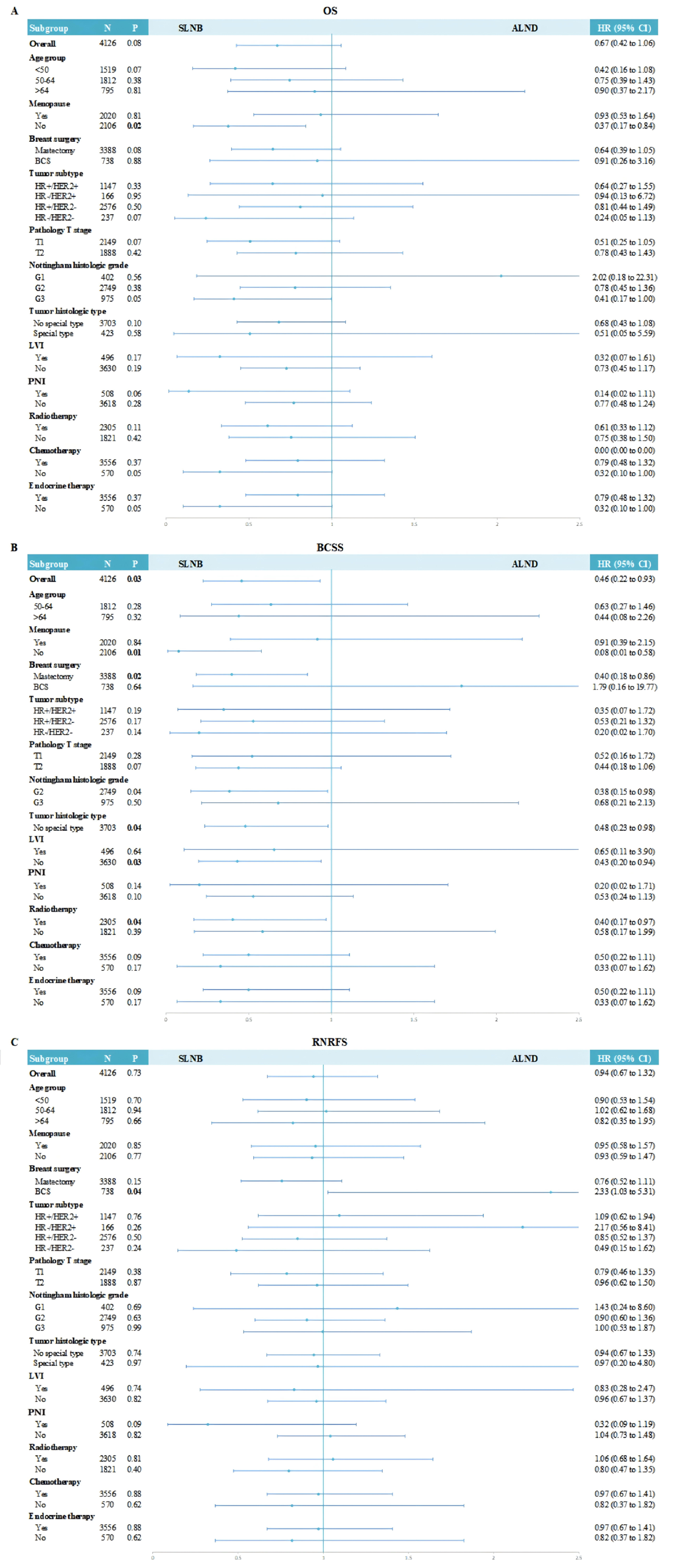

Supplement: Supplementary Figure S5 — Subgroup analyses of survival outcomes in the matched pN1 cohort. Forest plots depict hazard ratios (HR) comparing sentinel lymph node biopsy (SLNB) versus axillary lymph node dissection (ALND) for (A) overall survival (OS), (B) breast cancer-specific survival (BCSS), and (C) regional nodal recurrence-free survival (RNRFS). [file Image5.jpeg]
